# Development and validation of a new Multidisciplinary Approach Competency Scale for Prevention of Child Abuse from Pregnancy (MUSCAT)

**DOI:** 10.1371/journal.pone.0249623

**Published:** 2021-04-06

**Authors:** Chika Sakakida, Etsuko Tadaka, Azusa Arimoto

**Affiliations:** 1 Tsurumi Welfare and Health Center, Yokohama City Office, Yokohama, Kanagawa, Japan; 2 Department of Community Health Nursing, Graduate School of Medicine, Yokohama City University, Yokohama, Kanagawa, Japan; Erasmus Medical Center, NETHERLANDS

## Abstract

Child abuse remains a major global problem. A high-quality multidisciplinary approach involving different professionals for the early prevention of child abuse beginning from pregnancy is paramount because child abuse is associated with multiple potential risk factors at individual and societal levels. A multidisciplinary approach to preventing child abuse involves interprofessional coordination, and requires clear definitions of professional competency. However, no scale to measure professional competency for such multidisciplinary approaches is available. This study aimed to develop and validate the Multidisciplinary Approach Competency Scale for Prevention of Child Abuse from Pregnancy (MUSCAT). First, a draft scale comprising 30 items was developed based on a literature review, and then refined to 21 items through expert interviews. Next, a cross-sectional survey was conducted among experts from 1,146 child and maternal health institutions (health centers, perinatal medical centers/hospitals, child consultation centers, midwife clinics, and kindergartens) in 10 major prefectures and cities throughout Japan. The questionnaire collected respondents’ demographic data and information about one child abuse case, and asked respondents to apply the provisional MUSCAT to the reported case. Finally, three of the 21 items were excluded by item analysis, leaving 18 items for exploratory factor analysis. Confirmatory factor analyses identified 10 items on two factors: “Collaborative Networking” and “Professional Commitment.” The goodness of fit index was 0.963, adjusted goodness of fit index was 0.939, comparative fit index was 0.988, and root mean square error of approximation was 0.043. The Cronbach’s alpha for the entire scale was 0.903, and values for the subscales were 0.840–0.875. The overall scale score was positively correlated with the Interprofessional Collaboration Competency Scale. The MUSCAT demonstrated acceptable internal consistency and validity, and has potential for use in advancing individual practice and team performance in multidisciplinary approaches for early prevention of child abuse.

## Introduction

Child abuse refers to the physical abuse, sexual abuse, emotional ill-treatment, and neglect of children under age 18 years [1]. Globally, an estimated 41,000 children under age 15 years are reported to lose their lives annually because of abuse, although the actual number is thought to be higher [1]. Child maltreatment develops in the context of a relationship of responsibility, trust, and authority between caregivers and children, and can result in manifest or latent harmful effects on a child’s health, survival, development, and dignity [2]. Child abuse is a major human rights and social welfare problem that can also affect health outcomes in adulthood [2].

In Japan, the Child Abuse Prevention Act was first enacted in 1933 and revised in 2000. In 1993, the background of child abuse was absolute poverty, but the issue is now widespread [3]. In 2017, the 210 local governmental child consultation centers across Japan received 122,578 consultations regarding child abuse. The majority of deaths related to child abuse (57.7%) were reported to occur in the child’s first year of life. This highlights the importance of preventive interventional health-risk management starting from before birth. Providing early help to children who are victims of or suspected to be at risk for child abuse can increase the opportunities for prevention, recovery, and healing [4].

Previous studies reported that there are both individual- and societal-level parental risk factors for child abuse. Individual parental risk factors include characteristics such as child’s and caregiver’s young age, marital status, mental health problems, high stress level, poor coping skills, history of childhood abuse, domestic violence, substance abuse, personality traits, knowledge of child development, parenting skills, and financial stressors (e.g., poverty and low socioeconomic level) [5–10]. Societal risk factors for parents include isolation, lack of social support, and living in an unsafe neighborhood [5–10]. Combined with a caregiver’s inability to manage stressors appropriately, these risk factors may lead to actualized child abuse.

Unidisciplinary approaches from professional groups such as public health [11], medical [12], welfare [13], education, and community volunteers [14] provide different perspectives on child abuse, and have been found to offer useful resources. However, the multiple potential individual and societal risk factors mean that high-quality multidisciplinary approaches for the early prevention of child abuse beginning from pregnancy are important. Moreover, high-risk caregivers often have complex problems that begin during pregnancy and continue through the puerperium and child rearing stages. Therefore, preventive interventional health-risk management provided by multidisciplinary experts who can support pregnant women and their children is necessary [15].

Various conceptual frameworks have been proposed for multidisciplinary approaches [16–19]. However, these approaches are broad and do not focus on preventing child abuse. Leutz [16] described three main stages or levels based on the strength of the multidisciplinary approach. The first step is connecting people, the second is coordination, and the third involves comprehensive integration. Preventing child abuse using a multidisciplinary approach requires these three steps. For example, in the first step, high-risk caregivers are connected with the necessary services, and professionals recognize each other’s roles and build basic relationships for a preventive approach. In the second step, an information-sharing and care-management system should be established for high-risk caregivers, which can then be systematically implemented among specialists from multiple health services. In the third stage, multi-occupation professionals act comprehensively, receive good referrals based on team members identifying and meeting caregivers’ changing needs, manage all care, and control or directly provide care across all key settings [16]. This process is similar to how professionals at the same organization work across facility barriers.

An essential factor in promoting a multidisciplinary approach is the competency of each professional. Barr [20] noted that there are three basic types of competencies related to multidisciplinary coordination ability. The first type is complementary competencies that can be differentiated within each specialized profession. The second type comprises common competencies that are required by all specialized occupations. For example, making diagnoses and selecting treatment are complementary competencies among physicians, whereas common competences are values shared across medical, public health, and welfare fields, as well as the ability to communicate with patients and service users. The third type is collaborative competency, in which a professional is required to cooperate with other specialized professionals. Barr [20] suggested that having these three competencies generated smooth coordination and collaboration between specialized professions.

Moreover, competency in a multidisciplinary approach for preventing child abuse requires perspectives and strategies for primary, secondary, and tertiary prevention of child abuse. Primary prevention of child abuse focuses on specific prevention by detecting high-risk caregivers at the susceptibility stage starting from pregnancy and intervening before child abuse occurs. Secondary prevention of child abuse focuses on early identification and provision of timely, effective interventions with caregivers at the earliest stages of child abuse. Tertiary prevention of child abuse focuses on recovery and prevention of recurrence for caregivers in cases of child abuse, and for their children in the post-abuse stage. From a practical perspective, these three types of prevention often overlap in child abuse cases. Therefore, a multidisciplinary approach for child abuse is needed across all prevention stages. It is also important that each stage is correlated with the other stages.

Existing multidisciplinary approach scales have been found to be valid and reliable tools for use in general clinical practice [17, 18], and for children with medical complexity [21]. However, no validated scale is available for multidisciplinary approaches to child abuse prevention starting during pregnancy. Early support starting from the pregnancy period and extending without interruption throughout the puerperal and childcare periods is important, especially for pregnant women who have a particular need for support to prevent child abuse (including fetal abuse and domestic abuse). Early intervention from pregnancy is effective in preventing and reducing the incidence of child abuse [22–24]. However, the focus should not be limited to pregnant women, but extend to cases where support is started after childbirth for mothers with babies and children. In addition, while targeting high-risk caregivers, it is important to consider the entire family, including babies, children, and fathers. Therefore, a scale is needed that covers professionals who work with parents of babies and children. Identification of competencies for multidisciplinary approaches to support high-risk caregivers with complex problems starting from pregnancy, along with the development of a scale to measure these competencies could promote multidisciplinary specialization. In turn, this could improve the quality of life for pregnant women who are at high risk for engaging in child abuse as well as that of their children. However, a scale that measures the competencies necessary for multidisciplinary approaches has yet to be developed. Therefore, this study aimed to develop the “Multidisciplinary Approach Competency Scale for Prevention of Child Abuse in Pregnant Women” (MUSCAT) and evaluate its reliability and validity.

## Materials and methods

### Study 1: Developing the scale

The conceptual framework and item pool for the MUSCAT was based on relevant literature and expert reviews. For the literature review, we identified studies in which the main concept or theme concerned “collaboration” and “prevention of child abuse in pregnant women.” We searched PubMed (1946–2018) and Ichushi-Web (1970–2018). The search terms were: “pregnancy,” “disadvantaged mother,” “child abuse,” “multidisciplinary,” “interdisciplinary,” “cooperation,” “collaboration,” “partnership,” “integration,” “linkage,” “relation,” “team,” “community,” “measurement,” and “scale.” From a total of 37 identified articles, a pool of 30 draft items was created based on: i) the process of interprofessional collaboration for the prevention of child abuse in pregnant women, ii) adaptation of these items for multiple professionals (rather than specific groups of professionals), and iii) practical usefulness of the items within the community health setting. Finally, draft items were chosen by the present authors and several researchers according to specific selection criteria. After repeated consultation, we decided that items drawn from scales used in previous studies were acceptable if they were sufficiently related to at least one of the points mentioned above (i–iii).

The pool of 30 draft items was reviewed through interviews with four professionals and four researchers. This review assessed the content validity, face validity, and practical usefulness of these items within the community health setting. Participating professionals included a medical doctor, a public health nurse, a social worker, and a kindergarten teacher; these professionals had over 5 years of professional experience with child abuse cases. The participating researchers were professors with previous and concurrent accomplishments regarding the topic of child abuse and scale development in the fields of community health nursing or public health. These experts were asked for feedback on the scale name, subject, and comprehensibility of each of the 30 items, and any other points that needed revision. We then revised the wording of each item based on feedback from these experts (e.g., avoiding double-barreled questions, avoiding indefinite words, making the item intention clear). Consequently, the draft 30-item MUSCAT was refined to include 21 items.

### Study 2: Validating the scale

#### Study participants

A cross-sectional survey was conducted with experts from 1,146 institutions for child and maternal health: health centers (n = 230), perinatal medical centers/hospitals (n = 269), child consultation centers (n = 93), midwife clinics (n = 82), and kindergartens (n = 472). These facilities were selected using systematic random sampling from publicly available information lists in 10 major prefectures and cities throughout Japan (Hokkaido, Saitama, Chiba, Tokyo, Kanagawa, Shizuoka, Aichi, Osaka, Hyogo, and Fukuoka). MacCallum et al. [25] highlighted the importance of the level of item communality in determining the sample size. With a sample size of more than 200 participants, the factor structure should be stable provided communalities are ≥.50 and factors are well-determined (at least three items per factor and items strongly loaded to factors) [25]. The response rate was assumed to be 18% with reference to a previous study on multidisciplinary collaboration for children in Japan [21]. Factor analysis requires data from more than 200 samples, meaning more than 1,100 potential participants were needed for this study.

Before distributing the questionnaires to participating institutions, we identified the relevant sample size for each institution from the open database. The chosen prefectures and cities covered nearly half of relevant institutions in Japan. Inclusion criteria for participating in this study were: 1) professional experience of interprofessional collaboration for prevention of child abuse (including fetal abuse) from pregnancy among high-risk caregivers with multiple risk factors and 2) being a member of a defined professional group (representative person) for their institution or section. The professional groups selected were: public health nurse (health center), social welfare worker (health center), doctor (medical center/hospital), nurse (hospital), child welfare worker (child consultation center), midwife (midwife clinic), and kindergarten teacher (kindergarten). The subjects of this study were high-risk caregivers with multiple individual and societal risk factors who started early support in the pregnancy period and were recognized as having a particular need for support to prevent abuse without interruption throughout the puerperal and childcare periods. The cases reported in this study were from the pregnancy period to about 1 year after giving birth, and covered pregnant women, babies, and children.

#### Measures

Respondents were asked to provide their demographic characteristics, including sex, age, main qualifications, years of work experience, affiliated institution, and whether they had experience supporting the prevention of child abuse by interacting with pregnant women.

Next, respondents were asked to answer two evaluation questions for each MUSCAT item. The first item used a 4-point Likert-like scale to evaluate how the importance of the MUSCAT item (0 = Not important, 1 = Not important to a certain extent, 2 = Important to a certain extent, 3 = Important). The second question used a 4-point Likert-like scale to evaluate whether the item was related to cases of obvious child abuse (0 = Disagree, 1 = Disagree to a certain extent, 2 = Agree to a certain extent, 3 = Agree). To improve the goodness of fit for these answers, respondents were asked to provide demographic characteristics of abuse cases, including sex, age, pregnancy and childbirth history, support start time, risk situation, and professionals involved in the collaboration before completing the MUSCAT. Those who had experienced multiple cases were asked to report one abuse case in which support for prevention of abuse was required from the pregnancy period and cooperation with multiple professional groups was necessary. This information was considered to reflect more proficient and capable responses as respondents’ level of experience in providing support increased. We used this information to examine the characteristics of respondents and reported cases.

To assess the convergent validity of the MUSCAT, respondents also completed the Interprofessional Collaboration Competency Scale for Children with Medical Complexity (ICC-CMC) [21]. This instrument measures advancements in individual professional practice and team performance in interprofessional collaboration for children with medical complexity. The scale comprises 12 items (e.g., “I share information with other professionals about the child’s and family’s understanding of the disease and symptoms”), with responses on a 4-point Likert-like scale (0 = Disagree, 1 = Disagree to a certain extent, 2 = Agree to a certain extent, 3 = Agree). The constructs covered by the ICC-CMC are “sharing needs assessment skills,” “resource development skills,” and “creative networking skills.” The ICC-CMC is a multidisciplinary ability evaluation scale for children and their caregivers who are receiving treatment at home in the community [21]. The total score ranges from 0 to 36, with higher scores indicating greater competency. This scale has a Cronbach’s alpha of 0.933, and has been shown to be valid and correlated with the level of interprofessional collaboration in the community [21]. We used this scale because the content was relevant to the MUSCAT in terms of caregivers, involvement of professionals from various fields, and development in community settings. The ICC-CMC can also be used to evaluate multidisciplinary collaboration with general children and caregivers. The scale authors suggested the ICC-CMC could be used to explore well-being and community development of all children and caregivers living in an area [21]. Therefore, with the agreement of the original authors, we considered the scale could assess multidisciplinary cooperation for general children and caregivers. In addition, healthcare professionals must identify and support children who need medical care from pregnancy as well as parents of children who need medical care that become pregnant, as a child’s need for medical care may contribute to abuse. The percentage of professionals with experience of providing support for pregnant women is considered high, but no scales were found that measured interprofessional collaboration for people working with at-risk parents/families in general. Therefore, we used the ICC-CMC as a measure of professional collaboration for parents/families of children with medical care needs, as the ICC-CMC measurement concept was the most similar to the MUSCAT among existing scales.

#### Ethical considerations

The Institutional Review Board of the Medical Department of Yokohama City University approved this study on July 23, 2018 (No. A180700006). All respondents provided written informed consent.

#### Statistical analysis

All analyses were conducted with IBM SPSS Statistics version 25.0 (IBM Corp. Armonk, NY, USA) and Amos 25.0 (Chicago, IL, USA). As a first step, item analysis and exploratory factor analyses were conducted to evaluate the reliability and convergent validity of the MUSCAT. The criteria for item analysis [26] included pass efficiency (average score <2.0 points), rate of response difficulty (unknown and non-respondents: ≥5%), distribution (ratings of “Important to a certain extent”/“Important” by <90% of the sample), good-poor analysis (no significant differences between the highest and lowest scoring groups), and item-total analysis (correlation coefficient: <0.300). In accordance with item-response theory, when measuring constructs that did not measure knowledge, we replaced item difficulty with a term that described the degree of characteristics necessary for the respondent to endorse that item [27]. The percentage of respondents who did not endorse each item was calculated when evaluating the difficulty of the questionnaire [27].

As a second step, we examined the remaining items through exploratory factor analysis (principal factor analysis) with promax rotation. The optimal number of factors was determined using eigenvalues and a scree plot. Item loadings were required to exceed 0.400. Factor reliability was determined using a Cronbach’s alpha ≥0.700 [28], and construct validity was verified with confirmatory factor analysis.

Model fit was examined with the goodness of fit index (GFI), adjusted GFI (AGFI), comparative fit index (CFI), and root mean square error of approximation (RMSEA). The model was accepted if the GFI, AGFI, and CFI were ≥0.900 and the RMSEA was ≤0.050. Correlation analysis was used to evaluate the criterion-related validity of the confirmed version of the MUSCAT with the ICC-CMC, with a correlation ≥0.700 considered adequate [29, 30]. Cronbach’s alpha was used to evaluate the internal consistency of the confirmed version of the MUSCAT, with a value ≥0.700 considered adequate.

## Results

### Respondents’ characteristics

Table 1 shows respondents’ demographic characteristics. From 1,146 potential respondents, 280 (24.2%) responses were received, and 276 (98.5%) valid questionnaires were included in the analyses (excluding those that did not complete the demographic characteristics items). Respondents were from health centers (34.4%), kindergartens (23.4%), and hospitals (19.0%).

**Table 1:** Respondents’ demographic characteristics.

		n or mean±SD	% or (range)
Sex	Female	246	90.4
(n = 272)	Male	26	9.6
Age of pregnant mothers, years		46.8±11.4	(23.0–82.0)
(n = 259)	<30	18	6.9
	30–39	45	17.4
	40–49	89	34.4
	50–59	75	29.0
	60–69	25	9.7
	70–79	6	2.3
	≥80	1	0.4
Main qualification	Public health nurse	111	40.7
(n = 273)	Midwife	40	14.7
	Childcare worker	36	13.2
	Teacher	25	9.0
	Social worker	23	8.4
	Doctor	15	5.5
	Child welfare worker	10	3.7
	Nurse	5	1.8
	Mental healthcare worker	2	0.7
	Other	6	2.2
Years of work experience		19.8±11.6	(1.0–60.0)
(n = 273)	<10	61	22.3
	10–19	78	28.6
	20–29	81	29.7
	30–39	44	16.1
	≥40	9	3.3
Affiliated institution	Health center	94	34.4
(n = 273)	Kindergarten	64	23.4
	Hospital	52	19.0
	Child consultation center	27	9.9
	Midwife clinic	19	7.0
	Other	17	6.2
Number of support experiences		70.7±20.1	0–200
(n = 235)	<10	105	44.7
	10–49	74	31.5
	50–99	15	6.4
	≥100	41	17.4

Missing data were excluded from each analysis.

SD, standard deviation.

Table 2 shows the demographic characteristics of reported cases. The most common basic situations needing support were economic distress (46.3%) and single mothers (39.3%).

**Table 2:** Demographic characteristics of reported cases.

		n or mean±SD	% or (range)
Age of pregnant women, years			
(n = 229)	<16	3	1.3
	16–19	33	14.4
	20–24	44	19.2
	25–29	44	19.2
	30–35	42	18.3
	36–39	23	10.0
	≥40	5	2.1
Number of pregnancies			
(n = 202)	1	76	37.6
	2	54	26.7
	3	27	13.4
	4	19	9.4
	5	9	4.5
	≥6	17	4.9
Number of births	0	58	27.1
(n = 214)	1	62	29.0
	2	39	19.3
	3	25	11.7
	4	20	9.3
	5	4	18.7
	≥6	6	2.8
Support start time	<11 weeks gestation	21	10.4
(n = 201)	11–15 weeks gestation	31	15.4
	16–19 weeks gestation	13	6.4
	20–24 weeks gestation	19	9.5
	25–29 weeks gestation	21	10.4
	30–34 weeks gestation	19	9.5
	≥35 weeks gestation	15	7.5
	>6 weeks after birth	40	20.0
	6–9 weeks after birth	10	5.0
	≥10 weeks after birth	12	6.0
Risk situation	Economic distress	106	46.3
(n = 229)	Single mother	90	39.3
	Mental illness	89	38.9
	Problem with childcare	84	36.7
	Social isolation	80	34.9
	Intellectual disability	69	30.1
	Abuse victim	50	21.8
	Unplanned pregnancy	43	18.8
	Teenager	39	17.0
	Drug use	4	1.7
	Alcohol use	2	0.9
Number of collaborating professionals		4.8±6.5	(1.0–20.0)
(n = 229)			

Missing data were excluded from each analysis.

SD, standard deviation.

### Item analysis

Table 3 shows the item analysis. Three items (11, 16, and 20) were excluded because the correlation between the items was >0.700; therefore, they were too similar to other items. This left 18 items for factor analysis.

**Table 3:** Initial version of the Multidisciplinary Approach Competency Scale for Prevention of Child Abuse from Pregnancy (N = 279).

		Pass efficiency^a^	Item difficulty^b^	Population distribution^c^	Inter-item correlation^d^	Good-poor analysis^e^	Item-total correlation (r)^f^		Exclusion
Item No.	Item								
1	I know the names and faces of other professionals involved in supporting a high-risk caregiver and child.	2.6±0.6	5.0	94.7	-	0.000	0.445	**	
2	I’m able to unhesitatingly ask professionals from other disciplines for advice related to supporting a high-risk caregiver and child.	2.7±0.5	3.2	98.1	-	0.000	0.671	**	
3	I’m able to take part in child abuse prevention work in the spirit of protecting the rights of the child.	2.8±0.4	5.0	98.9	-	0.000	0.634	**	
4	I’m able to appropriately manage the private information of a high-risk caregiver and child.	2.9±0.4	3.2	98.9	-	0.000	0.540	**	
5	I’m able to share community resources available to a high-risk caregiver and child with other professionals.	2.7±0.5	3.9	97.4	-	0.000	0.742	**	
6	I’m able to promptly share information across professional disciplines when a person is determined to be a high-risk caregiver in need of support.	2.8±0.5	1.8	98.2	-	0.000	0.755	**	
7	I understand the need for trusting relationships between professionals and high-risk caregivers and children.	2.8±0.5	6.0	97.7	-	0.000	0.634	**	
8	I have a clear understanding of the role of each professional on the child abuse prevention team.	2.6±0.5	3.5	96.6	-	0.000	0.704	**	
9	I’m able to participate in building the abuse prevention safety net needed by a high-risk caregiver and child.	2.5±0.6	7.1	95.7	-	0.000	0.707	**	
10	I’m able to communicate to other professionals what has been done and what support has been provided to a high-risk caregiver and child as well as what outcomes have been accomplished.	2.7±0.5	3.2	97.4	+	0.000	0.785	**	
11	I’m able to share information with other professionals about problems that could happen in the future that would affect the daily lives of a high-risk caregiver and child.	2.8±0.4	2.1	99.6	+	0.000	0.818	**	**×**
12	I’m able to share with other professionals the future aspirations a high-risk caregiver has for her life.	2.6±0.6	3.9	94.4	-	0.000	0.749	**	
13	I’m able to explain to the caregiver the community resources available to her and her child.	2.7±0.5	3.9	97.8	+	0.000	0.680	**	
14	I understand that abuse prevention includes the need to collaborate and adjust support services among the different professions during quiet times.	2.7±0.5	4.6	98.5	-	0.000	0.682	**	
15	I’m able to set up a system for reporting, communicating, and consulting with those in the other professions to enable emergency abuse prevention responses.	2.8±0.4	2.5	99.3	+	0.000	0.781	**	
16	I’m able to make sharing issues with other professionals related to the support of a high-risk caregiver and child possible.	2.8±0.4	2.5	99.3	+	0.000	0.827	**	**×**
17	I try to reach agreement with other professionals about support objectives and plans for a high-risk caregiver and child.	2.7±0.5	3.2	98.1	+	0.000	0.798	**	
18	I understand the importance of the need to maintain continuity of support when a high-risk caregiver and child move.	2.9±0.4	2.8	99.3	-	0.000	0.681	**	
19	I’m able to reflect on my own work to support a high-risk caregiver and child and identify issues with my own professional expertise.	2.6±0.6	5.3	96.2	+	0.000	0.703	**	
20	I’m able to reflect on the abuse prevention support team’s work with a high-risk caregiver and child and identify collaboration issues.	2.7±0.6	3.2	96.7	+	0.000	0.685	**	**×**
21	I’m able to appropriately cope with interprofessional conflict regarding support for a high-risk caregiver and child.	2.5±0.6	7.4	94.6	+	0.000	0.713	**	

** *p* < 0.001, × Excluded items.

Exclusion criteria for the item analyses

^a^ Average score under 2.0 points (1 = Not important; 2 = Not important to a certain extent).

^b^ Percentage of “I don’t know” and NA: >5% of the sample.

^c^ Percentage of “Important” and “Important to a certain extent”: <90% of the sample.

^d^ Correlation: >0.7 (+).

^e^ Difference in average score between the highest and lowest scoring groups: No significant difference (*p* < 0.001).

^f^ Correlation coefficient between the item and the total of all items (excluding that item) <0.3.

### Exploratory factor analysis

The exploratory factor analysis results are shown in Table 4 (n = 229). After inputting 18 items, one item (item 1) with a factor loading <0.400 was excluded. Of the 17 items that were reintroduced, two items (items 5 and 10) with a factor loading <0.400 were excluded. Next, 15 items were input and three items (items 2, 13, and 18) with a factor loading <0.400 were excluded. Finally, 12 items were input; two items (items 9 and 19) with a factor loading <0.400 were excluded, and an optimum two-factor solution was obtained with 10 items. The Cronbach’s α value improved to >0.900 after excluding these items. Factor 1 (“Collaborative Networking”) included five items that explained the mutual connections of relationships and collaboration between multiple professional groups. Factor 2 (“Professional Commitment”) included five items that explained the responsibilities and involvement during support and care, which reflected the importance of awareness as professionals. The cumulative contribution of the two factors explained 64.9% of the variance. The correlation coefficient for the two factors was 0.888, and the Cronbach’s alpha coefficients were 0.875 for Factor 1, 0.840 for Factor 2, and 0.903 for the total scale.

**Table 4:** Exploratory analyses of the Multidisciplinary Approach Competency Scale for Prevention of Child Abuse from Pregnancy (final version) (n = 229).

Cronbach’s alpha coefficient		Factor 1	Factor 2	Total
		0.875	0.840	0.903
Initial version scale item no.		[Collaborative Networking]	[Professional Commitment]	Communality
21	I’m able to appropriately cope with interprofessional conflict regarding support for a high-risk caregiver and child.	0.846	−0.044	0.455
17	I try to reach agreements with other professionals about support objectives and plans for a high-risk caregiver and child.	0.785	−0.109	0.575
8	I have a clear understanding of the role of each professional on the child abuse prevention team.	0.719	0.126	0.501
15	I’m able to set up a system for reporting, communicating, and consulting with those in the other professions to enable emergency abuse prevention responses.	0.696	0.054	0.603
12	I’m able to share with other professionals the future aspirations a high-risk caregiver has for her life.	0.662	0.130	0.548
7	I understand the need for trusting relationships between professionals and high-risk caregivers and children.	−0.014	0.763	0.504
4	I’m able to appropriately manage the private information of a high-risk caregiver and child.	−0.124	0.727	0.373
6	I’m able to promptly share information across professional disciplines when a person is determined to be a high-risk caregiver in need of support.	0.036	0.655	0.59
3	I’m able to take part in child abuse prevention work in the spirit of protecting the rights of the child.	0.203	0.638	0.442
14	I understand that abuse prevention includes the need to collaborate and adjust support services among the different professions during quiet times.	0.181	0.598	0.511
Cumulative contribution (%)		54.2	64.9	
Factor correlation coefficients (r)	Factor 1	1.00		
	Factor 2	0.888**	1.00	

** *p* < 0.001. Principal factor analysis with promax rotation.

### Internal consistency and validity of the final scale

**Fig 1:**
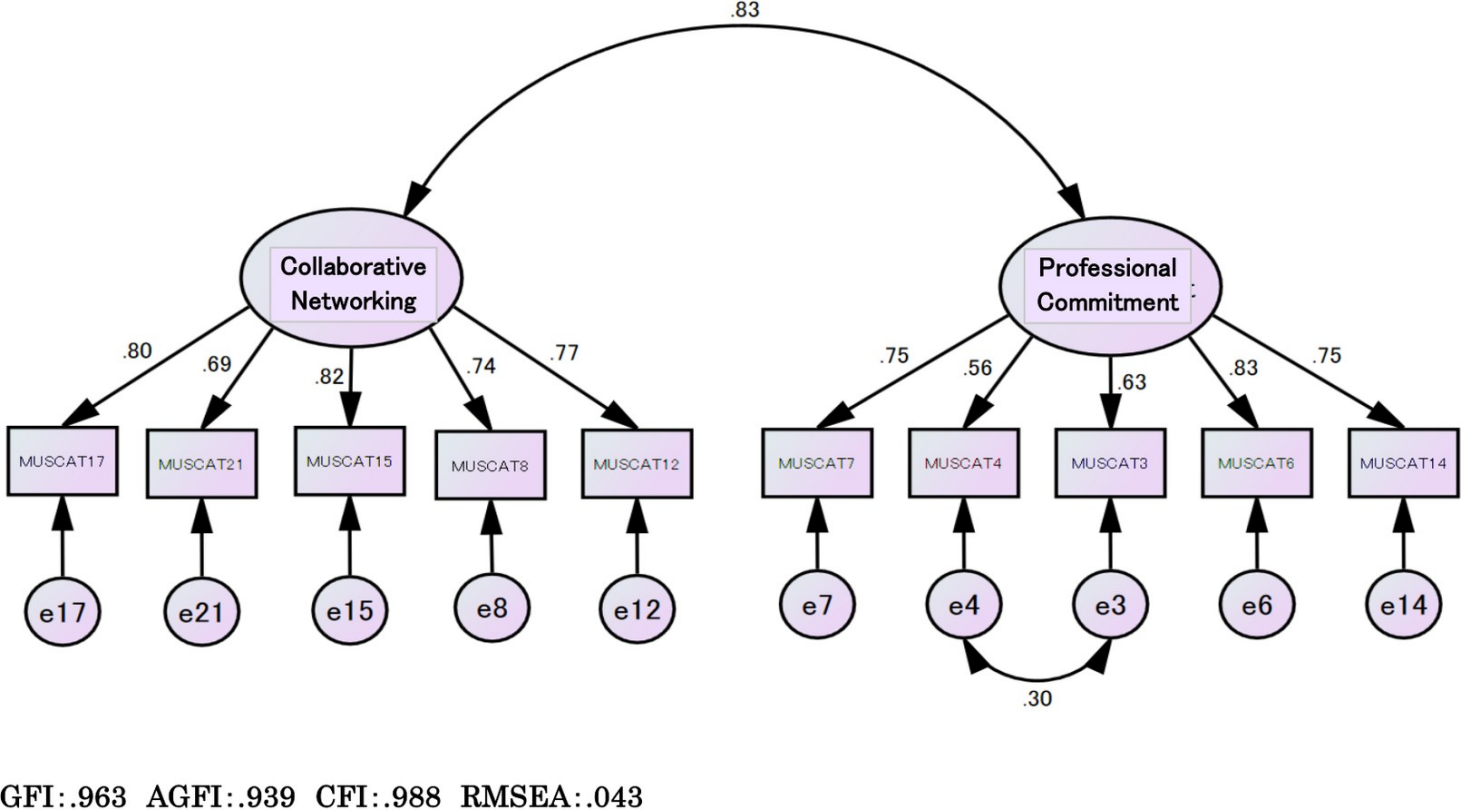
Confirmatory factor analysis for the Multidisciplinary Approach Competency Scale for Prevention of Child Abuse (final version).

The confirmatory factor analysis results are shown in Fig 1. The confirmatory factor analysis was performed using the same sample as used for the exploratory factor analysis. The model fit indices showed a GFI of 0.963, AGFI of 0.939, CFI of 0.988, and RMSEA of 0.043, which nearly satisfied the appropriate criteria for all respondents. We found moderate significant correlations between the two MUSCAT factors and the ICC-CMC: 0.552 for Factor 1, 0.478 for Factor 2, and 0.565 for the total scale (*p* < 0.01).

## Discussion

The results of our study suggested that the MUSCAT may be a valid and reliable scale to assess healthcare professionals’ competency in terms of a multidisciplinary approach for prevention of child abuse from pregnancy. The MUSCAT is based on self-reflection by professionals or multidisciplinary teams and is therefore similar to previous interprofessional collaboration scales [31, 32]. However, the MUSCAT has two novel aspects. First, while existing multidisciplinary approach scales target children living at home, care recipients, and medical care personnel [19, 21], the MUSCAT targets at-risk caregivers with complex risk factors for child abuse (including fetal abuse) from pregnancy, with consideration of the entire family (i.e., fetus, babies, children, and fathers). Second, compared with existing multidisciplinary approach scales that assess cooperation, the concept of “Professional Commitment” revealed in this study (focused on keeping “the child first” in mind) is an original and novel idea. However, there is no concept in the current scale that indicates responsibility in terms of protecting the rights of the child. The MUSCAT is a measure that can comprehensively evaluate healthcare professionals’ competency in relation to providing a multidisciplinary approach, from primary to tertiary prevention. Competences evaluated by the MUSCAT can also be developed through study or experience, and may increase as specialized work experience increases.

The first factor in the MUSCAT was “Collaborative Networking,” which can be considered a competency that is required for support and making adjustments in initiating, developing, and maintaining relationships with multiple professional groups. In optimal professional collaboration, each professional should understand other professional’s roles and draw on each other’s knowledge and skills, while at the same time fulfilling their own professional role. This type of collaboration requires adjustment and coordination. Objectives, governance, and framework-related decisions have been shown to have the same level of importance for achieving more coordinated work processes [33]. Management of child protection requires the combined skills and resources of different agencies working together; the quality of interagency collaboration has a direct impact on partnerships between agencies and families. Partnership is about services, sharing, information, accountability, and communication [30]. The construction of reliable networks is indispensable for developing relationships characterized by coordinated work processes [36]. These concepts result in frameworks that enable professionals to share information with caregivers and children and facilitate agreement on support policies and plans. In addition, each professional repeatedly responds appropriately to conflicts that sometimes arise between professional types, which reflect on their own profession. By engaging in formal and informal networking with other parties, professionals can develop specialized initiatives, support each other through networking, and establish long-term and mutually beneficial relationships [33].

The second MUSCAT factor, “Professional Commitment,” represented competency in reflecting on the ideas, behavior, emotions, and values of one’s own profession, as well as collaborating with multiple professional groups while maintaining a deep understanding of the coordination experience, and using this competency in collaboration and coordination efforts. Moreover, the principle of all professionals “working together to protect the child” lies at the center of child protection [34–37]. Engaging responsibly in support as a professional while remaining constantly aware of the rights of each child is an important characteristic in abuse prevention. The United Nations Convention on the Rights of the Child (1989) offers such a framework. The Convention affirms the principle that the child’s interests should be the primary consideration, and the state holds a special duty not to harm children. Therefore, child protection must be rooted in the twin principles that are of primary importance to the child, and minimal intrusion into family life. Professionals also have responsibility to seamlessly link with other professional groups if they judge that there is a high risk for abuse.

The MUSCAT may contribute to improving the ability of individual professionals to cooperate, as it can be used for evaluation and capacity development of multidisciplinary approaches in training programs. This will support improvement of individuals’ multi-job collaboration capability and development of multidisciplinary cooperation according to the actual conditions of the area. Furthermore, this may contribute to improving the quality of life of caregivers, children, and the wider community. The scale can also be used by team members (who have differing job categories) to develop self-reflection and think about the kind of contribution they can make through multidisciplinary approaches. Personal self-evaluation will lead to reflection. Moreover, all team members may develop their multi-occupational collaborative ability by using this scale [34]. There are many types of professions that may be involved in multi-occupational collaboration for early (i.e., during pregnancy) prevention of child abuse, including doctors, nurses, public health nurses, midwives, social workers, child welfare workers, kindergarten teachers, and nursery school teachers. Self-evaluation can confirm necessary skills and promote multidisciplinary approach through upgrading individuals’ skills. The MUSCAT can also be used by teams. When a high-risk caregiver case occurs, each individual professional in a team can evaluate and clarify the abilities related to offering a multidisciplinary approach that need to be improved to support that case, and share this information with the team. Therefore, each professional and the entire team can improve the capacity for multidisciplinary approaches.

This study had several limitations. First, the response rate was not as high as reported in previous related studies, which might have introduced bias into the survey results. Further studies will need to test for non-response effects to maximize validity. Second, because the study design was cross-sectional, it could not reveal the causal relationship between the MUSCAT and collaboration practices or outcomes. Therefore, a longitudinal design is needed to determine the predictive validity of the MUSCAT. The MUSCAT was developed for self-evaluation of individual abilities. In general, scales for self-evaluation are detailed, accessible, and easy to administer and interpret. However, there is a possibility that professionals’ abilities differ depending on job types or other related factors. Therefore, it is necessary to study other potential factors related to the competency for multidisciplinary approaches according to job type or ability. Finally, we conducted exploratory and confirmatory factor analyses with the same sample. It is desirable to use separate samples or randomly divide participants into two groups and perform one type of factor analysis with each sample. However, we judged that this would be difficult to achieve and result in small samples that would be difficult to analyze.

## Supporting information

S1 Appendix MUSCAT English version. (PDF)

S2 Appendix MUSCAT Japanese version. (PDF)
