# Supplementary material for: Development and validation of a new Multidisciplinary Approach Competency Scale for Prevention of Child Abuse from Pregnancy (MUSCAT)
Source: PLoS One. 2021 Apr 6;16(4):e0249623. doi: 10.1371/journal.pone.0249623 (PMC8023485; doi:10.1371/journal.pone.0249623)
Supplement: S1 Appendix — (PDF) [file pone.0249623.s001.pdf]

S1 Appendix

MUSCAT : English versions of the Multidisciplinary Approach Competency Scale for Prevention of Child Abuse from Pregnancy

Please evaluate it.

Agree : 3
Agree to a certain extent : 2
Disagree to a certain extent : 1
Disagree : 0

No	Factor & Item	evaluation			
**Factor 1**	**Collaborative Networking**				
1	I'm able to appropriately cope with interprofessional conflict regarding support for a high-risk caregiver and child.	3	2	1	0
2	I try to reach agreements with other professionals about support objectives and plans for a high-risk caregiver and child.	3	2	1	0
3	I have a clear understanding of the role of each professional on the child abuse prevention team.	3	2	1	0
4	I'm able to set up a system for reporting, communicating, and consulting with those in the other professions to enable emergency abuse prevention responses.	3	2	1	0
5	I'm able to share with other professionals the future aspirations a high-risk caregiver has for her life.	3	2	1	0
**Factor 2**	**Professional Commitment**				
6	I understand the need for trusting relationships between professionals and high-risk caregivers and children.	3	2	1	0
7	I'm able to appropriately manage the private information of a high-risk caregiver and child.	3	2	1	0
8	I'm able to promptly share information across professional disciplines when a person is determined to be a high-risk caregiver in need of support.	3	2	1	0
9	I'm able to take part in child abuse prevention work in the spirit of protecting the rights of the child.	3	2	1	0
10	I understand that abuse prevention includes the need to collaborate and adjust support services among the different professions during quiet times.	3	2	1	0
	No.1～10 total	point			

Sakakida C, Tadaka E, Arimoto A : Development and Validation of a New Multidisciplinary Approach Competency Scale for Prevention of Child Abuse from Pregnancy (MUSCAT) . PLOS ONE, 2021
doi:10.1371/journal.pone.0249623
