# Supplementary material for: Development and validation of a new Multidisciplinary Approach Competency Scale for Prevention of Child Abuse from Pregnancy (MUSCAT)
Source: PLoS One. 2021 Apr 6;16(4):e0249623. doi: 10.1371/journal.pone.0249623 (PMC8023485; doi:10.1371/journal.pone.0249623)
Supplement: S2 Appendix — (PDF) [file pone.0249623.s002.pdf]

S2 Appendix

MUSCAT-J ： Japanese versions of the Multidisciplinary Approach Competency Scale for Prevention of Child Abuse from Pregnancy

日本語版妊娠期からの虐待予防のための多職種連携能力評価尺度

各項目について、評価してください。

領域	No	項目	3 あてはまる	2 ややあてはまる	1 ややあてはまらない	0 あてはまらない
Ⅰ 協働的ネットワーキング	1	ハイリスク養育者・子どもへの支援に関する多職種間の葛藤に対し適切に対処できる	3	2	1	0
	2	ハイリスク養育者・子どもの支援方針・計画について他職種と合意を図れる	3	2	1	0
	3	虐待予防支援チームにおける各専門職の役割分担を明確に認識できる	3	2	1	0
	4	虐待予防にむけた緊急時の対応を可能にするための他職種との報告・連絡・相談体制を整えられる	3	2	1	0
	5	ハイリスク養育者の将来における生活の希望を他職種と共有できる	3	2	1	0
Ⅱ 専門的コミットメント	6	ハイリスク養育者・子どもと専門職における信頼関係の必要性についてわかる	3	2	1	0
	7	ハイリスク養育者・子どもに関する個人情報を適切に管理できる	3	2	1	0
	8	ハイリスク養育者の可能性がある対象者と判断した場合、速やかに多職種と情報共有できる	3	2	1	0
	9	子どもの権利擁護の精神にのっとり、虐待予防に携わることができる	3	2	1	0
	10	虐待予防にむけた平時の多職種間での連携・調整の必要性についてわかる	3	2	1	0
		No.1～10 合計				点

Sakakida C, Tadaka E, Arimoto A：Development and Validation of a New Multidisciplinary Approach Competency Scale for Prevention of Child Abuse from Pregnancy (MUSCAT). PLOS ONE,2021
doi:10.1371/journal.pone.0249623
